# Shoulder Arthroplasty: Historical Considerations

**DOI:** 10.2174/1874325001711011100

**Published:** 2017-09-30

**Authors:** Sébastien Zilber

**Affiliations:** Henry Mondor Teaching Hospital 51 ave Mal de Lattre de Tassigny 94010 Créteil, France

**Keywords:** Shoulder, Arthroplasty, History, Shoulder prosthesis

## Abstract

**Background:**

The first articular metal prosthesis was implanted in the shoulder more than 120 years ago. The aim of this paper is to report shoulder arthroplasty evolution during this time thru the literature of the twentieth century.

**Methods:**

A literature review was performed selecting the founding papers about shoulder arthroplasty.

**Results:**

After being almost forgotten during the first part of the 20^th^ century, various implants were introduced in the 1950s with Charles Neer as a leader. The reverse concept appeared in the 1970s and knew many failures before Grammont’s design.

**Conclusion:**

After many unfortunate trials, the shoulder prosthesis is now widely disseminated with products of many companies.

## 1 INTRODUCTION

It is little known that the first articular metal prosthesis ever implanted was likely a total shoulder prosthesis. The procedure was carried out in Paris (France) by surgeon JE Péan for the treatment of tuberculosis of the shoulder [1]. Various shoulder arthroplasty designs were attempted with varying degrees of success until the Neer [2] anatomical and the Grammont [3] reverse concepts became the two gold standards. The first metal shoulder prosthesis [4] Jules Emile Péan (1830-1898), a French surgeon, implanted a constrained total shoulder prosthesis made of platinum and rubber for the treatment of a patient with shoulder tuberculosis arthritis in 1893 [1]. Using Themistocle Glück’s schematics [5], Péan first developed an ivory prosthesis that was never implanted out of concern of its poor mechanical properties and poor biocompatibility. A new prosthesis was then constructed by a Parisian dentist who specialized in prosthetic development, Dr. JP Michaels. The stem was made of platinum with screw holes at the distal end for attachment to the humeral bony stump. The head consisted of a rubber ball with metal loops inserted into a groove for attachment to the glenoid and to the proximal aspect of the stem.

The patient was a 37 year old male dying of tuberculosis of the right shoulder and proximal humerus. He refused amputation, so Péan resected the proximal half of the humerus and removed infected tissue. The prosthesis was implanted at a second intervention. The prosthesis allowed mobility such that « the patient was using his arm for most of the daily activities » [1]. An elbow fistula appeared one year later that required four drainage procedures. At 2 years, in the face of a persistent fistula, radiographs demonstrated an osseous shell surrounding the prosthesis. The sepsis was successfully treated with removal of the prosthesis.

The prosthesis was donated to the Armed Forces Institute of Pathology in the United States in1916 and is now on display in the Smithsonian Institution in Washington. This was probably the first metal articular prosthesis ever implanted as Themistocle Glück [5], the pioneer of joint replacement, was using ivory and cadaveric bone for his prostheses.

## 2 PLASTIC IMPLANTS IN THE 1950S

Various implant descriptions were published in the 1950s beginning with plastic prostheses. These were made of acrylic [6, 7], polyamide [8] or of polyethylene [9].

Richard [6] was the first to use this type of implant. He implanted humeral head acrylic prostheses for complex proximal humerus fractures which were typically treated by bone resection [7]. Although it was suggested as an alternative to bone resection, active mobility was often poor when the great tuberosity was resected even if the infraspinatus and supraspinatus were reattached to the acrylic humeral head Fig. (**1**). MacAusland [8] also used a plastic prothesis made of polyamide to treat a comminuted fracture dislocation of the proximal humerus with encouraging early results.

Four massive polyethylene proximal humeral prostheses were implanted at the Royal National Orthopaedic Hospital in the United Kingdom for reconstruction following tumor resection. The implants were fixed with plates and screws to the distal humerus. All failed due to screw pull-out of the bone [9]. One prosthesis fractured.

These plastics prostheses were eventually abandoned due to breakages and attrition caused foreign body reactions [10].

## 3 METAL IMPLANTS SINCE 1950

The first modern metal hemiarthroplasty was heralded by Krueger [11]. An anatomically shaped metal prosthesis made of chrome-cobalt alloy (vitallium) was implanted for the treatment of avascular necrosis of the humeral head. The prosthesis was implanted with the preservation of the rotator cuff insertions to bone; the result was a well functioning and painless shoulder.

Charles Neer’s early efforts in the development of shoulder prostheses were directed at patients with poor function and persistent pain following humeral head resection for fracture of the neck of the humerus with dislocation of the head fragment [2]. The first Neer prosthesis was implanted in 1953 [12]. According to Neer, shoulder arthroplasty design features should include material that is inert and strong with an elasticity close to the bone, preservation of normal anatomy, and sufficient anchorage with a long stem and large interface to avoid bone resorption. Neer reported a series of eight shoulder hemiarthroplasties, the “Neer 1 prosthesis”, made of vitallium for the treatment of fracture-dislocations, avascular necrosis, and a single case of osteoarthritis with very encouraging results [12]. This represented the first well-designed shoulder prostheses, and Neer stressed the importance of tuberosity fixation and healing. As results were not satisfactory in cases with a defective rotator cuff, Between 1970 and 1972, Neer went on to design the “ Averill 3“ fixed-fulcrum prostheses with a reversed glenohumeral articulation. He concluded that a fixed fulcrum design failed to compensate for a deficient rotator cuff and was at high risk for mechanical failure [13]. Engelbrecht [14] and Kenmore [15] had independently designed polyethylene glenoid components for use with the Neer 1 prosthesis. Neer recognized that a total shoulder replacement might improve the functional results when the glenoid was arthritic. He went on to develop the “Neer 2” system that was the first to have multiple humeral and glenoid components designed for use together in a nonconstrained total shoulder [13]. The Neer 2 system did address the lower functional results that occurred in case of cuff deficiency however.

Lettin and Scales reported on two cases of total shoulder replacement using the Stanmore prosthesis to treat rheumatoid arthritis in 1972. Both resulted in pain relief and achieved 90° of abduction, 6 months and 1 year following the procedure Fig. (**2**) [16]. In 1982, the authors published the results of 49 Stanmore total shoulder replacements performed between 1969 and 1977 [17]. Nine patients were left with an excision arthroplasty (one due to infection and another recurrent dislocation) and glenoid loosening that occurred between one month and 2 years following replacement in the remainder. The functional improvement for the remaining 40 patients was inconsistent and disappointing according to the authors, although an acceptable functional range of motion was achieved in most patients. Similar to the Bickel shoulder prosthesis [18], the Stanmore prosthesis maintained the standard ball-and-socket gleno-humeral articulation, although with increased constraint.

The “Michael Reese” prosthesis described by Post in 1975 was a standard, constrained ball-and-socket gleno-humeral articulation [19]. The humeral component was modified 2 years after the first implantation due to complications of bending and breakage of the humeral neck. Post reported disappointing gain in function and complications such as dislocation and glenoid loosening in a series of 102 prostheses [20]. The authors suggested that prior to using a constrained prosthesis, other less extensive shoulder reconstructions should be considered.

Swanson used a bipolar shoulder implant that was designed in 1975 [21]. This consisted of a hemiarthroplasty with a large unfixed metal glenoid cup and a polyethylene liner that articulated with a small ball of the humeral titanium cemented stem. The main indication for the implant was in severely arthritic shoulders with rotator cuff arthropathy.

In 1977, Mazas described a nonconstrained total shoulder prosthesis using a full glenoid polyethylene flat cup cemented to the glenoid and the acromion and a cemented humeral metal stem [22]. Five years later, the results of 38 cases were published [23]. It was often necessary to resect the supraspinatus tendon in order to implant the humeral stem and a posterior approach through the infraspinatus and the teres minor was frequently used. . Fourteen revisions were performed for instability or glenoid loosening, and active mobility was disappointing in two thirds of cases.

The Dana shoulder prosthesis [24], the Roper-Day prosthesis [25], and the Custom shoulder prosthesis [26] based on the Neer 2 system as were many other total prostheses that have appeared since the eighties. Zippel was the first investigator to publish a report describing the use of a metallic humeral shell used to resurface the humeral head while articulating with a polyethylene glenoid component in 1975 [27]. Resurfacing became popular at the end of the twentieth century with good results largely published by Copeland (28).

## 4 REVERSE SHOULDER PROSTHESES

A shoulder reversed prosthesis was first described by Reeves in 1972 [29]. Various reversed constrained prostheses were described, with a small glenoid metallic sphere on a neck that reproduced an anatomic or lateralised center of rotation.

- The Kölbel prosthesis was intended for shoulder reconstruction after tumor resection [30]. Glenoid fixation was secured with a flange that was bolted to the base of the scapular spine or to the scapular pillar.
- The Kessel prosthesis [31] utilized a single large central glenoid screw. Like the Kölbel prosthesis, the humeral stem was made of polyethylene. In a series of 23 prostheses followed for at least 5 years, 6 revision surgeries were reported before 3 years of follow up, and radiolucent lines were observed around all glenoid components [32]. The design was improved by Bayley and Walker Fig. (**3**); the glenoid screw was coated with hydroxyapatite and the center of rotation was moved medially and distally [33]. The humeral stem was changed to metal with a polyethylene retentive liner.
- In 1973, Gerard [34] published the results of 6 implantations of reverse total shoulder prostheses, with a metal glenoid plate fixed with 2 screws in the scapula. and a hole in the center were the A 20 mm metal metal sphere was screwed into the plate. The humeral component consisted of a polyethylene semi-retentive cup fixed on a metal stem Fig. (**4**). Shoulder stability and pain relief was obtained in all patients. However, active movement did not improve, as the prosthesis design did not compensate for the rotator cuff.
- The Liverpool shoulder was initially designed in 1969 by Beddow and Elloy and was similar in design to a reversed hip prosthesis; the glenoid component and stem were cemented into the scapular pillar, and a polyethylene cup was cemented into the proximal humerus [35].

With the introduction of the large glenoid sphere, led to an improvement in deltoid function. The Fenlin prosthesis [36] consisted in a large polyethylene glenoid sphere which articulated with a large cup on a metallic humeral stem. Breakage, loosening and instability were observed at long-term followup [37]. Buechel introduced a double-mobility cup with a small metal glenosphere that articulated with a large polyethylene ball which in turn articulated with the humeral metal cup and stem to allow supraphysiologic motion [38]. The Gristina trispherical system was also designed to optimize mobility [39]. This constrained system included a small humeral metal ball and a small glenoid metal ball that both articulated with a large, central polyethylene sphere.

Grammont reported on his reversed system in 1987 [3]. The main innovation was to medialize the center of rotation of the glenohumeral articulation to increase deltoid function in a prosthesis that was inherently stable. His first version of the prosthesis consisted of a cemented metal glenosphere that made up 2/3 of a sphere. This articulated with a full polyethylene cemented humeral stem and cup that consisted of 1/3 of a sphere. After loosening and breakage of the glenoid component was encountered, the center of rotation was medialized vis à vis the native glenoid surface by altering the glenosphere from 2/3 of a sphere to ½ a sphere [40]. Both glenoid and humeral component were coated with hydroxyapatite for uncemented fixation. From the 1990s, the Grammont system was adopted by many shoulder surgeons for the treatment of cuff deficiency, as it was superior to all other systems.

## CONCLUSION

The shoulder prosthesis is now more than 120 years old. After many design iterations, the Neer and the Grammont concepts are the two gold standards. New implants offer the possibility to implant an anatomic or a reverse design on the same humeral stem or resurfacing humeral base Fig. (**5**).

## Figures and Tables

**Fig. (1):**
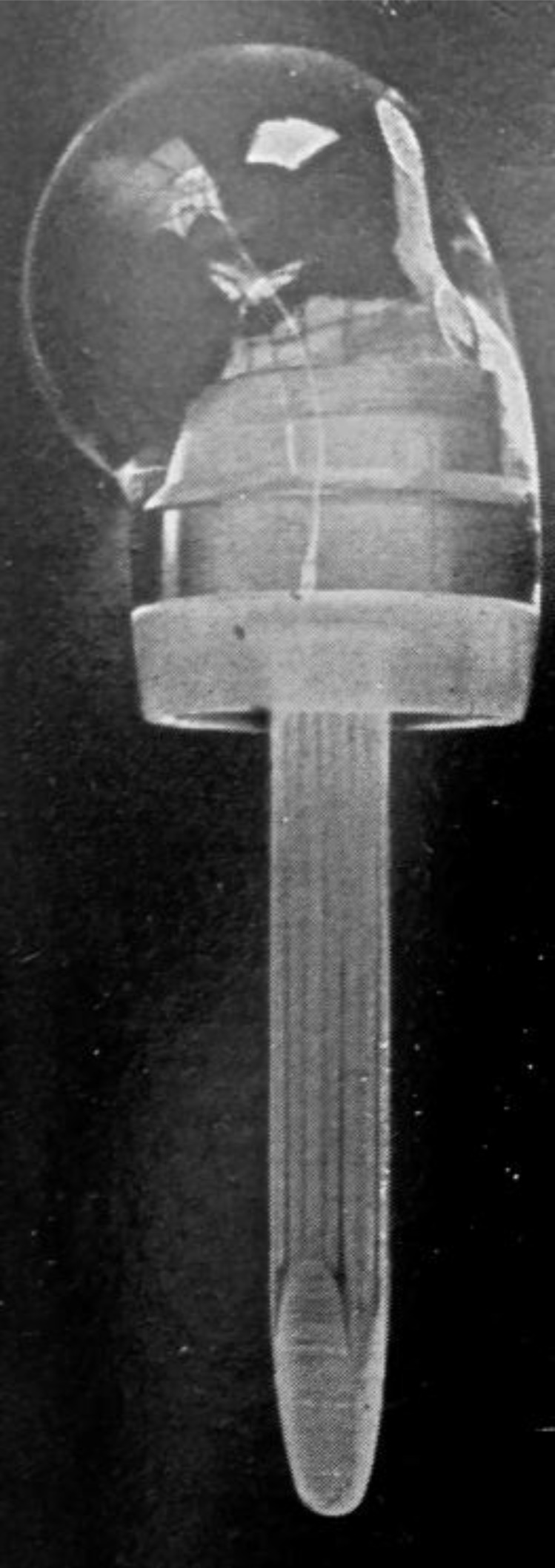
Richard acrilyc humeral prosthesis.

**Fig. (2):**
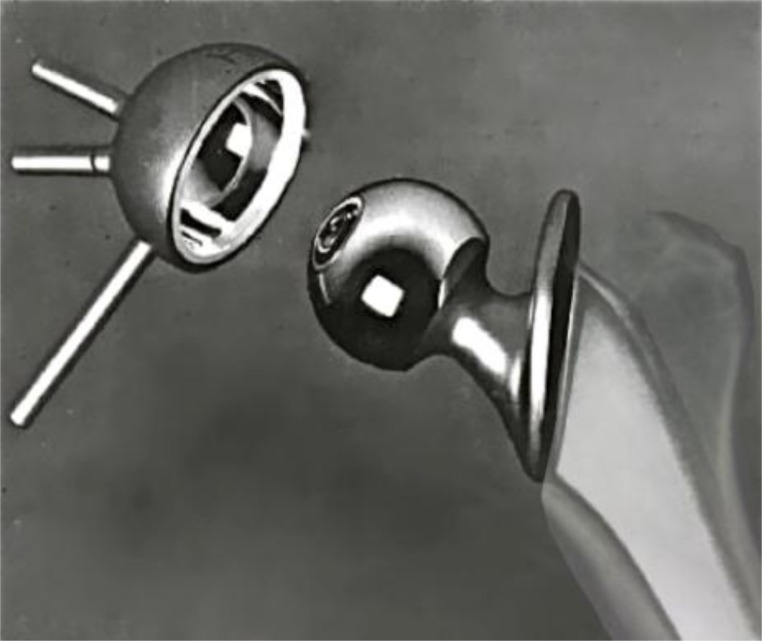
Stanmore prosthesis.

**Fig. (3):**
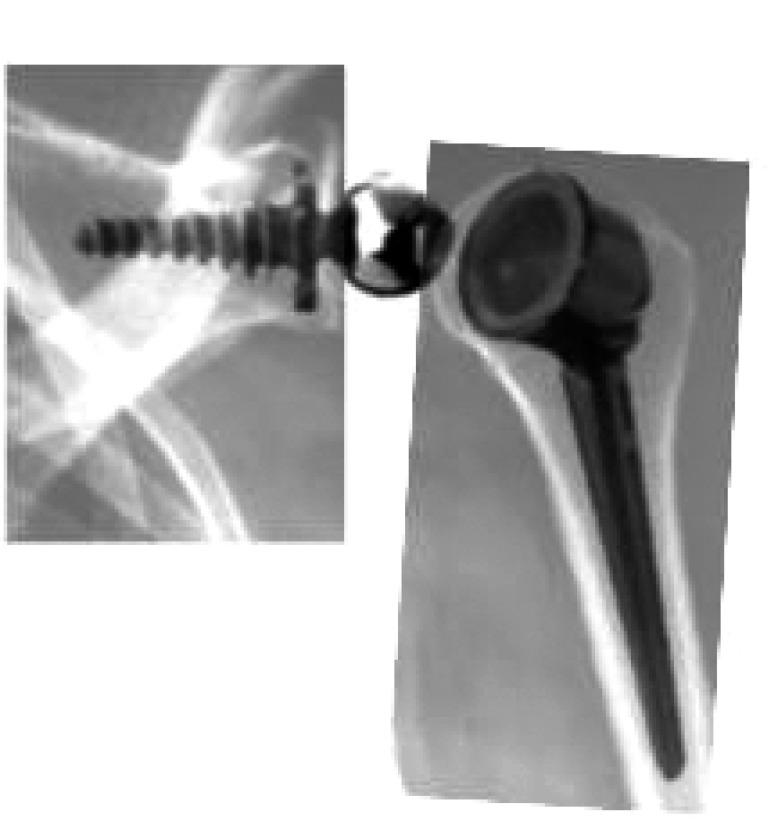
Bayley and Walker prosthesis (Kessel concept).

**Fig. (4):**
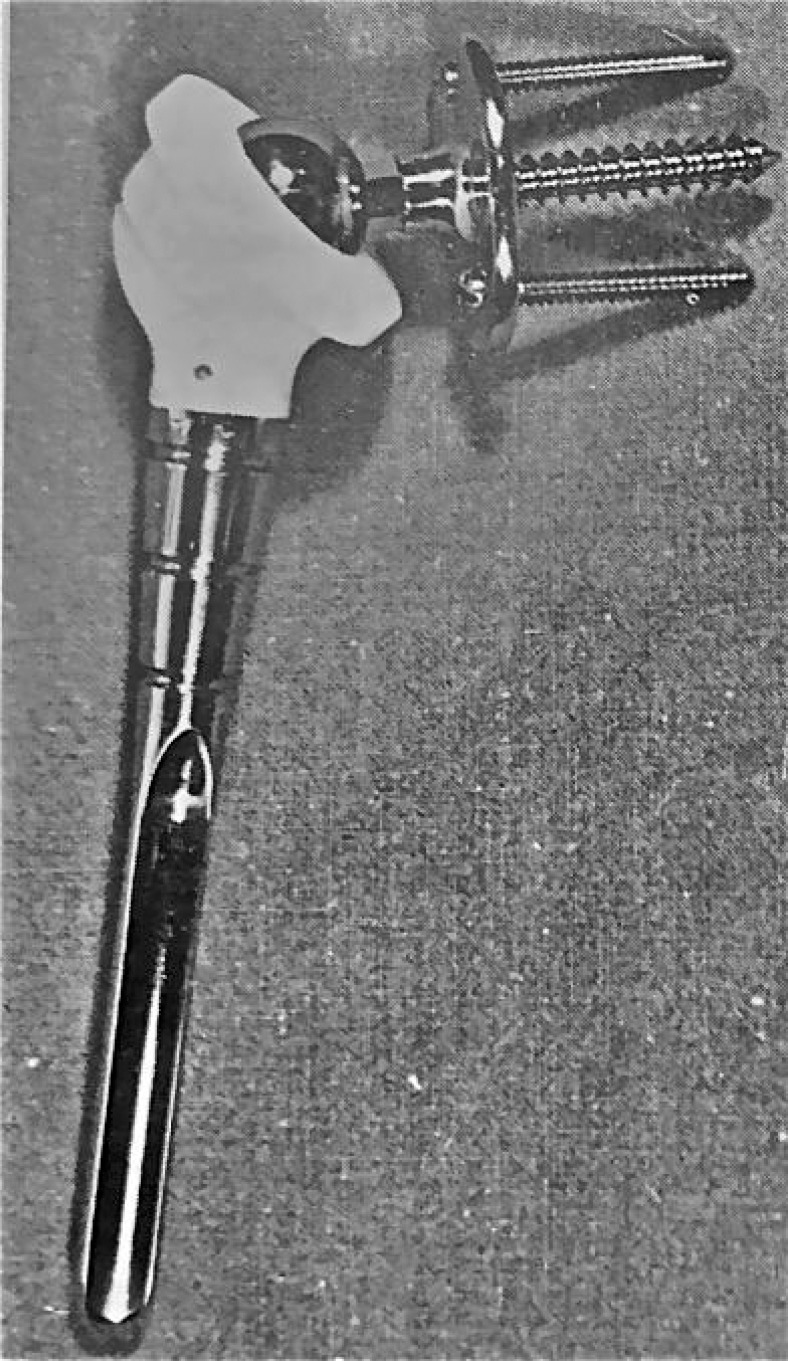
Gerard and Lannelongue prosthesis.

**Fig. (5):**
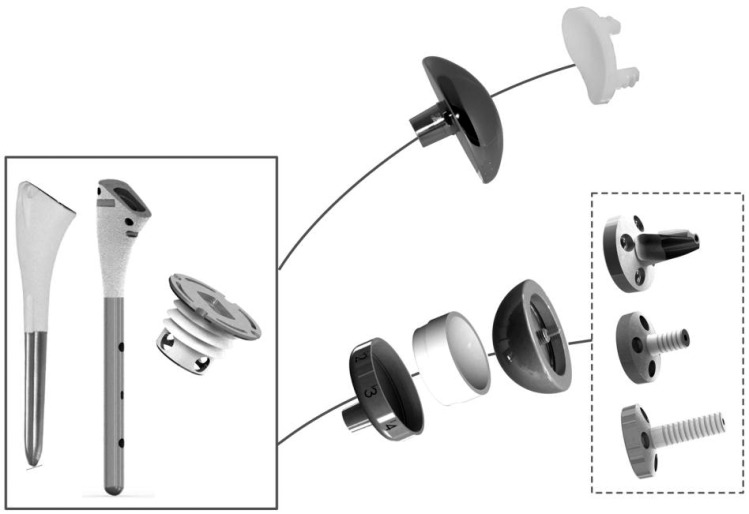
example of a contemporary platform stem implant (3S ORTHO)
